# Gun carrying among freshmen and sophomores in Chicago, New York City and Los Angeles public schools: the Youth Risk Behavior Survey, 2007-2013

**DOI:** 10.1186/s40621-018-0143-1

**Published:** 2018-04-10

**Authors:** Samaa Kemal, Karen Sheehan, Joe Feinglass

**Affiliations:** 1 Department of Pediatrics, Children’s Hospital of Philadelphia, Philadelphia, PA 19104 USA; 2 Injury Prevention and Research Center, Ann & Robert H. Lurie Children’s Hospital, Chicago, IL 60611 USA; 3 Division of General Internal Medicine and Geriatrics, Northwestern University Feinberg School of Medicine, Chicago, IL 60611 USA

**Keywords:** Weapon carrying, Gun carrying, Youth violence, Chicago

## Abstract

**Background:**

This study evaluated trends and risk factors over time for self-reported gun carrying among freshman and sophomore public school students in Chicago, New York City and Los Angeles, chosen as high profile cities with different levels of firearm violence.

**Methods:**

The study used four biennial waves (2007-2013) of the Youth Risk Behavior Survey (YRBS), an anonymous, voluntary survey of public high school students. Analyses were restricted to freshman and sophomores given significant high school dropout rates among older students. School population weighted results are presented based on the YRBS complex survey design, including comparisons of reported gun carrying across survey waves and cities. A violence index was created from eight survey items that capture students’ perceived threat level. Chi square tests and multivariable Poisson regression analyses were used to test the significance of differences across cities and over time in the likelihood of gun carrying controlling for sociodemographic characteristics, mental health risk factors and behavioral risk factors.

**Results:**

The study included a total weighted population estimate of 1,137,449 students across the three cities and four survey waves. Mean self-reported gun carrying across all survey waves was 8.89% in Chicago, 4.09% in New York City, and 6.03% in Los Angeles (*p* < 0.001). There were no significant changes in gun carrying prevalence within each individual city over the survey waves. Multivariable Poisson regression estimates showed increased likelihood for gun carrying among males (IRR 1.41, CI 1.27-1.58), among non-Hispanic Blacks (IRR 1.26, CI 1.07-1.48), and among those who reported a higher violence index. Each additional violence index count increase was associated with a 1.74 times (CI 1.70-1.78) increased likelihood for gun carrying.

**Conclusions:**

There was a much higher self-reported rate of gun carrying and a higher burden of violence exposure in Chicago as compared to New York City and Los Angeles. Students’ exposure to violence extended to other stressors illuminated by the YRBS including fighting, perceptions of safety, and other high-risk behaviors. Through the violence index we created, we are better able to categorize the most high-risk individuals and describe the magnitude of their increased likelihood to carry a gun.

## Background

Homicide is the second leading cause of mortality in youth aged 10-19 years with firearm-related homicides being the most common form (Web-based Injury Statistics Query and Reporting System (WISQARS) n.d.). The burden of violence disproportionately affects racial and ethnic minorities and otherwise at-risk disadvantaged youth. When homicides are stratified by race and ethnicity, homicide becomes the leading cause of mortality among African Americans and the fourth leading cause among Non-Hispanic Whites, revealing a significant racial disparity (Web-based Injury Statistics Query and Reporting System (WISQARS) n.d.). As of 2015, the Center for Disease Control and Prevention (CDC) reported 16.2% of high school students in the United States reported carrying a weapon in the last 30 days and 5.3% reported carrying a gun in the last 30 days (Kann 2016). Weapon carrying by youth not only places them at risk for committing violent acts but also increases the likelihood of morbidity and mortality from violence in other manifestations (Lewis et al. 2007b).

Our study uses four biennial waves of the Youth Risk Behavior Survey to advance understanding of gun carrying among youth by analyzing the characteristics of freshman and sophomore gun carriers in three major United States cities with a significant violence and homicide burden: Chicago, New York City and Los Angeles. We describe overall trends and differences in gun-carrying prevalence across these cities as part of a wider examination of students’ perceived risk of violence.

## Methods

This study used the Youth Risk Behavior (YRBS), a biennial anonymous, voluntary survey of high school students that is administered by school districts and supported by the CDC. The target population is all public and private school students in grades 9-12 in the 50 states and District of Columbia. The purpose of this survey is to monitor priority health risk behaviors that result in the most significant morbidity and mortality in adolescents. The de-identified and publicly available YRBS data were ruled exempt for review by the Northwestern University Institutional Review Board.

### Study participants

Each district employs a two-stage, cluster sample design in order to attain a representative sample of students in grades 9-12 in its jurisdiction. YRBS data is weighted to adjust for school and student nonresponse as well as oversampling of Black and Hispanic students. A scientifically drawn sample, proper documentation of the sampling process and an overall response rate ≥ 60% are all required to be reported as weighted data (Brener et al. 2013).

The Chicago Public Schools calculated five-year cohort dropout rates for our four survey years (2007, 2009, 2011, 2013) of 41.6%, 42.5%, 39.8% and 33.6% respectively (Chicago Public Schools Department of School Quality Measurement n.d.). Given the significant amount of dropout, we chose to narrow our analysis to include only 9th and 10th graders in order to try to capture as accurate a representation of students as possible.

### YRBS data from Chicago, New York City and Los Angeles

This study used the district-weighted data for Chicago, New York City and Los Angeles from the survey wave years 2007, 2009, 2011 and 2013. The inclusion of data over four survey years allowed for analysis of changing prevalence of behaviors over time while simultaneously increasing analytic sample size. Each district’s YRBS data is weighted to be representative of its jurisdiction. Table 1 presents the survey questions included in this study. Questions 1-9 were categorized as “violence index” variables. These variables (excluding #2) were used to develop a total violence count and then a violence index to numerically describe an individual’s exposure to and threat of violence. Each variable was assigned one point, and the violence count was calculated by summing the total number of factors for each individual, with a total of up to eight points possible. From the violence count, the violence index was developed by stratifying into violence index risk categories based on the total violence count. No violence was associated with a total violence count of zero, low violence was associated with a total violence count of one to three and high violence was associated with a total violence count of four or more.

**Table 1:** Youth Risk Behavior Survey questions

1. During the past 30 days, on how many days did you carry a weapon such as a gun, knife, or club?
2. During the past 30 days, on how many days did you carry a gun?
3. During the past 30 days, on how many days did you carry a weapon such as a gun, knife, or club on school property?
4. During the past 30 days, on how many days did you not go to school because you felt you would be unsafe at school or on your way to or from school?
5. During the past 12 months, how many times has someone threatened or injured you with a weapon such as a gun, knife, or club on school property?
6. During the past 12 months, how many times were you in a physical fight?
7. During the past 12 months, how many times were you in a physical fight on school property?
8. During the past 12 months, have you ever been bullied on school property?
9. During the past 12 months, have you ever been electronically bullied? (Count being bullied through e-mail, chat rooms, instant messaging, websites or texting).
10. During the past 12 months, did you ever feel so sad or hopeless almost every day for 2 weeks or more in a row that you stopped doing some usual activities?
11. During the past 12 months, did you ever seriously consider attempting suicide?
12. During the past 12 months, how many times did you actually attempt suicide?
13. During the past 30 days, on how many days did you smoke cigarettes?
14. During the past 30 days, on how many days did you have at least one drink of alcohol?
15. During the past 30 days, on how many days did you use marijuana?
16. During your life, how many times have you used a needle to inject any illegal drug into your body?
17. During the past 3 months, with how many people did you have sexual intercourse?

### Statistical analysis

Statistical analysis was performed using STATA Version 14 (Statacorp, College Station, TX). The svy module was utilized to account for complex survey weights across multiple survey years. Chi square tests were used to determine statistical significance of bivariate associations between gun carrying and the above variables related to other types of weapon carrying, violence exposure and additional sociodemographic and personal characteristics. Several multivariable Poisson regression models were developed including one using the total violence count and another using the violence index. The regression analyses were used to estimate the likelihood of being a gun carrier, controlling for respondents’ demographics, mental health and behavioral risk factor characteristics.

## Results

Our final analyses included 54,096 actual freshman and sophomore respondents corresponding to a total weighted study population across all four survey waves and cities of 1,137,449. Included in this are the weighted populations of Chicago (198,422), New York City (582,802), and Los Angeles (356,225) freshman and sophomore respondents. Characteristics of the YRBS respondents are shown in Table 2. There was a smaller weighted population in the sophomore group compared to the freshman group (527,919 vs. 609,529). Most students were from a racial/ethnic minority though the distribution varied considerably based on city. Chicago participants had a statistically significant (*p* < 0.05) higher prevalence of almost all mental health and behavioral risk factors.

**Table 2:** Sociodemographics characteristics and health risk factors YRBS weighted data for freshman and sophomores from 2007 to 2013

	Chicago *N* = 198,422	New York City *N* = 582,802	Los Angeles *N* = 356,225
Grade			
9th	49.69%	50.20%	52.92%
10th	50.31%	49.80%	47.08%
Sex			
Male	53.28%	52.59%	51.19%
Female	46.72%	47.41%	48.81%
Ethnicity			
Non-Hispanic White	6.68%	12.35%	9.39%
African Americans	39.49%	26.28%	4.93%
Hispanic	43.90%	42.34%	44.30%
Other/Unknown	9.94%	19.03%	41.37%
Health Risk Factors			
Ever feel sad or hopeless in last 12 months*	29.91%	27.12%	29.76%
Ever considered suicide in last 12 months*	14.12%	12.43%	13.93%
Ever attempted suicide in last 12 months**	10.08%	7.33%	7.88%
Smoked cigarettes in the last 30 days**	18.54%	12.39%	14.13%
Drank alcohol in last 30 days**	44.09%	34.53%	39.51%
Smoked marijuana in the last 30 days**	28.73%	18.38%	22.01%
Injected drugs ever**	7.41%	8.27%	4.50%
Greater than 2 sex partners in last 3 months**	29.75%	22.72%	16.59%

*p < 0.05

**p < 0.001

Gun carrying prevalence among freshman and sophomores across all cities was 5.53%. The self-reported frequency was much higher among males compared to females (8.44% vs. 2.54%, *p* < 0.001). When stratified by race and ethnicity, African Americans had the highest self-reported gun carrying (6.29%), followed by Other/Unknown (5.61%), followed by Hispanics (5.48%), followed by Non-Hispanic Whites (3.51%). Gun carrying among African American males in particular was 9.76%.

**Fig. 1:**
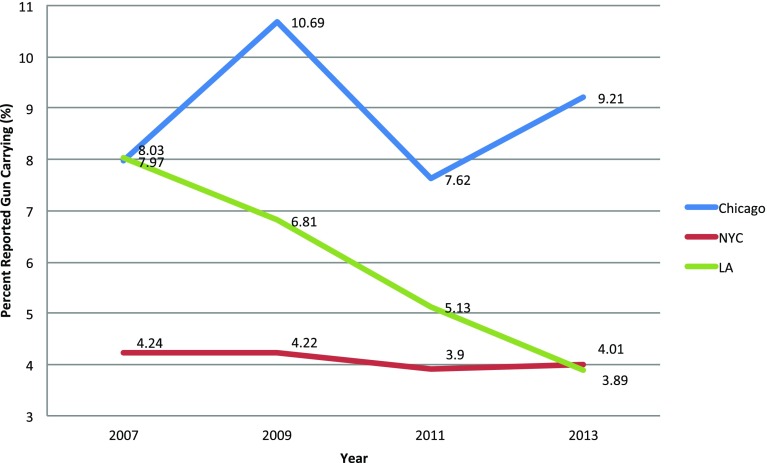
Trends in self-reported gun carrying among freshman and sophomores in Chicago, New York City and Los Angeles from 2007 to 2013 using YRBS weighted data. This figure depicts the trend analysis of self-reported gun carrying in Chicago, New York City and Los Angeles. Chicago maintained a higher prevalence than both Los Angeles and New York City following 2007. Los Angeles shows a steady decline over the 4 years while New York City maintains a steady rate over the 4 years. There was not a statistically significant difference within any of the three cities over this time period

Chicago had a higher prevalence in self-reported gun carrying among freshman and sophomores across the cumulative four survey waves (*p* < 0.001). Figure 1 depicts trends over time in gun carrying among the three cities. While there is no statistically significant difference within any of the three cities over the four survey waves, Los Angeles shows a steady decline while New York City maintains a steady rate over the 4 years. While Chicago and Los Angeles had similar gun carrying prevalence in 2007, the Los Angeles rate declined while the Chicago rate increased and remained higher than both other cities.

Table 3 shows the survey-weighted responses from items regarding weapon carrying and exposure to and fear of violence. Chicago had a higher prevalence of self-reported weapon carrying of all categories. Chicago additionally had a higher prevalence in most measures regarding fear of or exposure to violence. The no violence index (0 affirmative responses) was representative of 34.14% of the freshman and sophomores across cities. A low violence index (1-3 affirmative responses) was representative of 57.39% of the freshman and sophomores across cities. A high violence index (4 or more affirmative responses) was representative of 8.47% of the freshman and sophomores across cities. The prevalence of a high violence index was further stratified by city as shown in Table 3. Chicago had a much higher prevalence of high violence index respondents than both New York City and Los Angeles (11.24% vs. 8.18% vs. 8.48%, *p* < 0.001).

**Table 3:** Violence index by city YRBS weighted data for freshman and sophomores from 2007 to 2013

Violence Index Variables	Chicago *N* = 198,422	New York City *N* = 582,802	Los Angeles *N* = 356,225
Carried any weapon in the last 30 days**	21.11%	12.48%	14.95%
Carried gun in the last 30 days**	8.89%	4.09%	6.03%
Carried any weapon to school in the last 30 days*	7.94%	5.65%	5.82%
Feeling unsafe at school in last 30 days*	12.67%	9.53%	9.05%
Threatened or injured at school in last 12 months**	13.40%	7.56%	10.40%
In physical fight in last 12 months**	46.70%	35.94%	36.70%
In physical fight at school in last 12 months**	25.26%	17.07%	20.48%
Bullied at school in last 12 months*	13.07%	13.98%	16.81%
Electronically bullied in last 12 months	10.38%	11.72%	10.59%
Risk Category			
No violence (0 violence index variables)**	31.75%	25.66%	49.36%
Low violence (1-3 violence index variables)**	57.01%	43.25%	57.39%
High violence (4 or more violence index variables)**	11.24%	8.18%	8.47%

*p < 0.05

**p < 0.001

The initial multivariable Poisson regression analysis, controlling for respondents demographic, mental health and behavioral risk factor characteristics, showed a greater likelihood of gun carrying among males (IRR 1.41, CI 1.27-1.58), students in Chicago (IRR 1.22, CI 1.13-1.33), students in LA (IRR 1.15, CI 1.04-1.30), African Americans (IRR 1.26, CI 1.07-1.48), Hispanics (IRR 1.17, CI 1.10-1.36), Other Races/Unknowns (IRR 1.30, CI 1.08-1.58), those who reported carrying a weapon in the last 30 days (IRR 93.69, CI 67.83-129.42), those who reported carrying weapons to school in the last 30 days (IRR 1.14, CI 1.02-1.28), those who reported feeling unsafe at school in the last 30 days (IRR 1.22, CI 1.09-1.37), those who reported being threatened or injured at school in the last 12 months (IRR 1.36, CI 1.23-1.49) and those who reported being in a physical fight in the last 12 months (IRR 1.14, CI 1.00-1.30).

Table 4 shows the results of the multivariable Poisson regression analysis conducted using the total violence count in place of the individual violence index survey items. Each additional affirmative item a student answers on the violence index was associated with a 1.74 times greater likelihood of being a gun carrier (CI 1.70-1.78). When multivariable Poisson regression analysis was conducted using violence index categories, the high violence index category (> 3) conferred a 6.51 times increased likelihood for gun carrying compared to those in the low violence index category (IRR 6.51, CI 5.68-7.46).

**Table 4:** Poisson regression analysis of likelihood of being a gun-carrier using total violence count YRBS weighted data for freshman and sophomores from 2007 to 2013

	Incidence Rate Ratio (95% Confidence Interval) *N* = 1,137,449
Male	1.94 (1.73-2.18)
Female	Reference
Freshman	Reference
Sophomore	0.97 (0.85-1.09)
Chicago	1.60 (1.44-1.78)
Los Angeles	1.47 (1.26-1.72)
New York City	Reference
African American	1.43 (1.14-1.78)
Hispanic	1.30 (1.08-1.57)
Other Race/Unknown	1.30 (1.07-1.58)
Non-Hispanic White	Reference
Total Violence Count	1.74 (1.70-1.78)
Ever feel sad or hopeless in last 12 months	0.83 (0.75-0.92)
Ever considered suicide in last 12 months	0.80 (0.70-0.90)
Ever attempted suicide in last 12 months	1.10 (0.97-1.25)
Smoked cigarettes in the last 30 days	1.30 (1.15-1.47)
Drank alcohol in the last 30 days	1.74 (1.45-2.09)
Smoked marijuana in the last 30 days	1.19 (1.01-1.40)
Injected drugs ever	1.07 (0.94-1.20)
Greater than 2 sex partners in last 3 months	1.65 (1.44-1.90)

## Discussion

In this study, we found there were no significant changes in adolescent gun carrying within each individual city over the four YRBS study waves though certain trends were observed over time. Compared to New York City and Los Angeles, freshman and sophomores in Chicago had a statistically significant higher self-reported prevalence of gun carrying and most other violence index risk factors. There was a higher likelihood for gun carrying among males compared to females, among non-Hispanic Blacks, and among those who reported a higher violence index. Being part of the high violence index category (> 3) conferred a 6.5 times increased likelihood for gun carrying compared to those in the low violence index category and, if analyzed continuously, each additional violence index was associated with 1.74 times increased likelihood for gun carrying. The city with the highest percentage of individuals in the high violence index risk category was Chicago. While shootings continue to decline in New York City and Los Angeles, they have dramatically increased in Chicago and are marked by including a high proportion of adolescent victims and perpetrators (Gun Violence in Chicago 2016).

### Implications for the recent spike of violence in Chicago

In 2016 alone, Chicago experienced 762 homicides, a 57% increase from 2015 and a number that exceeded Los Angeles and New York City combined (Towers and White 2017). There is undeniably a multifactorial explanation for why Chicago has remained in the spotlight for high prevalence of violence and firearm-related homicides. The city has a deep history of segregation both socioeconomically and racially that has created a larger burden of need in specific pockets of the city. It is well known that violence is superimposed on our most disadvantaged communities, poor communities and communities of color, so it should not be surprising that the highest murder rates in Chicago are often in areas that are > 90% African American and high in poverty (Community Health Status Assessment n.d.). While segregation in other major cities such as New York City arguably decreased with gentrification and immigration, the segregation in Chicago only seems to be becoming more entrenched resulting in compounding hardships of poverty and crime (Musterd and Ostendorf 2013).

According to the Illinois Violent Death Reporting System (IVDRS) from 2008 to 2014, for individuals ages 10-24 in Cook County, the county Chicago falls under, the annualized age-adjusted death rate per 100,000 for firearm-related homicide was 20.85 compared to a national rate of 5.97 (Web-based Injury Statistics Query and Reporting System (WISQARS) n.d.). The variances became even more profound when further stratifying by race. In Chicago, the rates per 100,000 for African American males was 109.37; Hispanic males 26.07; Non-Hispanic White males 1.90 (Web-based Injury Statistics Query and Reporting System (WISQARS) n.d.). This shows that African American males aged 10-24 years experienced greater than 50× more firearm-related homicides than their Non-Hispanic White counterparts during this time period. For Los Angeles and New York City, the same type of data shows that the rates are overall much lower than Chicago though still slightly higher than the nationwide values (Web-based Injury Statistics Query and Reporting System (WISQARS) n.d.).

### Fear of violence and the toll on urban youth

The increased burden of violence in Chicago is not isolated to firearms and homicides but also extends further and is part of many other stressors illuminated by the YRBS including fighting, perceptions of safety, mental health concerns and other high-risk behaviors. The ability to formulate a total violence index using YRBS data provides a lens through which to evaluate violence prevention outreach efforts. Existing literature supports the interrelatedness of many of these factors (Stayton et al. 2011; Lewis et al. 2007a; Muula et al. 2008). The total violence count and violence index risk categories analyzed here help to show that the more factors a single individual takes on, the more likely they are to carry a gun and, we can hypothesize, the more likely they are to enter a cycle of violence. This guides us to a theory of clustering in which disadvantaged communities, often communities of color, are isolated in managing the compounding effects of segregation, poverty and crime concurrently without the resources available to others.

### Limitations

There was no weighted data available for the 2015 YRBS in Chicago, which prevented the ability to compare the three cities for this most recent survey year. This limited the ability to show the most up-to-date self-reported behaviors of students in the time period in which violence increased considerably in Chicago. Additionally, we know that there is a significant dropout rate in all three cities though dropout is particularly predominant in Chicago (Chicago Public Schools Department of School Quality Measurement n.d.; California Department of Education n.d.; New York Department of Education 2012). Students lost to drop out are not accurately represented in the study data and likely represent an important population to target. We attempted to mitigate the impact of this problem by restricting analysis to freshman and sophomores but, nonetheless, as evidenced by the decrease in weighted population from freshman to sophomore years, we undoubtedly lost important potential members of our study population to dropout. Finally, these data are self-reported and it is unknown how honestly students answer questions about firearms, however, it is unlikely that this would bias inter-city comparisons.

## Conclusions

This study supports much of what has already been shown in the existing literature regarding risk factors for gun carrying, but it is especially of interest to further understand why some cities are making significant progress while others, like Chicago, have not. We show the multiple layers of violence and fear of violence in which Chicago students outpaced other cities in 2007-2013. Through the violence index we created, we are better able to categorize the most high-risk individuals and describe the magnitude of their increased likelihood to carry a gun. Reducing youth violence will require a massive investment in educational opportunities, job creating, community renewal and revitalization.

## Abbreviations

CDC: Center for Disease Control and Prevention
CI: Confidence Interval
IRR: Incidence Rate Ratio
YRBS: Youth Risk Behavior Survey
